# Trends in Necrotizing Fasciitis‐Associated Mortality in the United States 2003–2020: A CDC WONDER Database Population‐Based Study

**DOI:** 10.1002/wjs.12504

**Published:** 2025-03-19

**Authors:** Saad Khan, Rizwan Ahmad, Aqsa Munir, Safa Nasir, Maryam Adnan, Fatima Naveed, Usama Idrees, Syeda Mashal Fatima, Javed Iqbal

**Affiliations:** ^1^ Saidu Medical College Khyber Pakhtunkhwa Pakistan; ^2^ Khyber Medical University Peshawar Pakistan; ^3^ Dow Medical College Karachi Pakistan; ^4^ Aga Khan University Hospital Karachi Pakistan; ^5^ Gujranwala Medical College Gujranwala Pakistan; ^6^ Rawal Institute of Health Sciences Islamabad Pakistan; ^7^ Khawaja Muhammad Safdar Medical College Sialkot Pakistan; ^8^ Nursing Department Communicable Diseases Center Hamad Medical Corporation Doha Qatar

**Keywords:** critical care, patient Safety, soft tissue infection

## Abstract

**Background:**

Necrotizing fasciitis (NF) is a severe and rapidly progressing soft tissue infection with high mortality rates. Despite the urgency of this condition, there is limited research on long‐term NF‐related mortality trends in the United States.

**Objective:**

This study aims to analyze NF‐related mortality trends in adults aged 25 and older in the United States from 2003 to 2020, focusing on variations by sex, race/ethnicity, and geographic region.

**Methods:**

**NF‐related deaths were identified using the CDC WONDER database** through the ICD‐10 code M72.6. Crude and age‐adjusted mortality rates (AAMRs) were calculated across demographic groups and regions. Temporal trends were assessed using the joinpoint regression, providing annual percent change (APC) in mortality rates.

**Results:**

From 2003 to 2020, a total of 19,158 NF‐related deaths were recorded, marking a 120.6% increase, rising from 824 deaths in 2003 to 1842 in 2020. The overall AAMR increased from 0.44 per 100,000 in 2003 to 0.71 per 100,000 in 2020. Males consistently had higher mortality rates than females and both sexes saw a sharp rise in AAMR after 2015. By race/ethnicity, American Indian or Alaska Native populations exhibited the highest mortality rates, followed by Black or African American individuals. Regional trends revealed that the West had the highest AAMR, whereas the Northeast recorded the lowest. A significant rise in mortality rates was observed across all regions after 2014. Additionally, urban–rural analysis indicated that large central metropolitan areas had consistently elevated mortality rates, whereas smaller metropolitan and noncore areas experienced sharper increases.

**Conclusions:**

NF‐related mortality has significantly risen in the United States since 2014, with distinct disparities based on sex, race, and geographic region. Contributing factors may include chronic conditions, healthcare access issues, and climate‐related events. Public health interventions focusing on early diagnosis, timely treatment, and addressing healthcare inequities are essential for improving outcomes (highlighted shows corrections).

## Graphical abstract

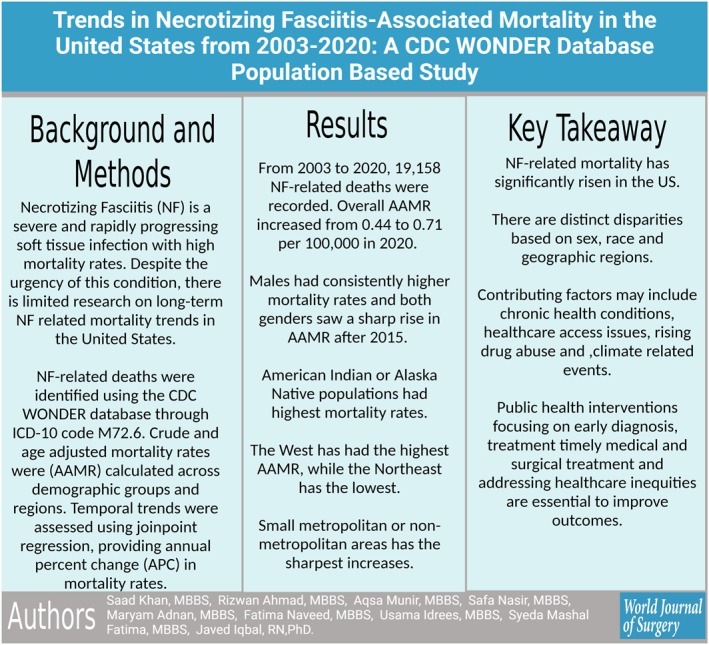

## 1 Introduction

Necrotizing fasciitis (NF) is a rare but life‐threatening soft tissue infection, marked by the rapid spread of inflammation and necrosis in the fascial planes and surrounding tissues. Although it often follows trauma, the initial trigger can be as minor as a scrape or insect bite. This condition is usually caused by virulent, toxin‐producing bacteria, particularly group A *Streptococcus*, and is associated with severe systemic toxicity. Without prompt diagnosis and aggressive treatment, necrotizing fasciitis can quickly become fatal, earning the nickname “flesh‐eating disease” due to its devastating effects on human tissue [1, 2]. NF is categorized into four subtypes based on the causative microorganisms, with the most common pathogens being *Staphylococcus aureus* and *Streptococcus* pyogens [3]. The cornerstone of treatment is immediate surgical debridement, paired with broad‐spectrum antibiotics [4]. Studies show that delays in diagnosis and treatment significantly raise the mortality rates linked to this critical condition [5].

The incidence of necrotizing fasciitis varies significantly, with rates ranging from 0.86 to 32.64 cases per 100,000 person‐years, influenced by factors such as climate and seasonal changes [6]. From 2003 to 2013, the mortality rate was 4.8 deaths per 1,000,000 person‐years [7]. Despite its rarity, the high mortality associated with necrotizing fasciitis underscores the need for heightened awareness and timely intervention, particularly among healthcare providers caring for high‐risk populations.

Despite growing concerns about the rising incidence of skin and soft tissue infections, there is limited data on long‐term trends in the epidemiology of necrotizing fasciitis (NF) in the United States [7]. This study will analyze NF‐related mortality trends among adults aged 25 and older from 2003 to 2020, examining crude and age‐adjusted mortality rates (AAMRs) across key demographic factors, such as sex, race/ethnicity, and urban–rural classifications. By investigating these trends, we aim to identify shifts in NF epidemiology, assess regional differences, and determine the populations most at risk. Understanding these patterns is crucial for guiding public health strategies, optimizing resource allocation, and developing targeted interventions (highlighted shows corrections).

## 2 Methods

### 2.1 Study Settings and Population

Our study utilized necrotizing fasciitis (NF)‐related mortality data from the CDC WONDER (Centers for Disease Control and Prevention Wide‐Ranging Online Data for Epidemiologic Research) database, encompassing the entire United States [8]. The study population included adults aged over 25. Data for NF‐related mortality were extracted for the study period 2003 to 2020 using the International Statistical Classification of Diseases and Related Health Problems (ICD‐10) code M72.6 for necrotizing fasciitis [8]. During data extraction, instances of necrotizing fasciitis were identified among the multiple causes of death. Since the study relied on publicly available data provided by the government, the institutional review board (IRB) approval was not required. All the data used in this study are publicly available at https://wonder.cdc.gov. The study follows STROBE guidelines for cross‐sectional studies [8] (highlighted shows addition).

### 2.2 Data Extraction

Data were extracted based on demographics, year, urbanization, population size, and region. The demographic categories included sex, age, and race/ethnicity. Race was classified into the following groups: non‐Hispanic (NH) White, NH Black or African American, Hispanic or Latino, NH American Indian or Alaskan Native, and NH Asian or Pacific Islander. Regions were categorized as Northeast, Midwest, South, and West, based on the 2013 Urbanization data. These included six groups: large central metropolitan areas, large fringe areas, medium metropolitan areas, small metropolitan areas, micropolitan areas, and noncore areas. This classification was derived from information available in the CDC Wonder database [9].

### 2.3 Statistical Analysis

To examine national trends in necrotizing fasciitis (NF)‐related mortality, we calculated crude and age‐adjusted mortality rates (AAMRs) per 100,000 population from 2003 to 2020. These rates were stratified by year, sex, race/ethnicity, state, and urban–rural status, with corresponding 95% confidence intervals (CIs). Crude mortality rates were derived by dividing the number of NF‐related deaths by the corresponding U.S. population for each year. AAMRs were calculated by standardizing NF‐related deaths to the 2000 U.S. standard population. The Joinpoint Regression Program (Joinpoint V 4.9.0.0, National Cancer Institute) was employed to determine the annual percent change (APC) in AAMRs, also with 95% CIs, to quantify national trends in NF‐related mortality as done in previous research [10, 11]. This method identifies significant changes in AAMRs over time by fitting log‐linear regression models to detect temporal variations. APCs were classified as increasing or decreasing based on whether the slope representing the change in mortality significantly differed from zero as assessed using the 2‐tailed t‐testing. A *p*‐value of less than 0.05 was considered statistically significant.

## 3 Results

Between 2003 and 2020, 19,158 mortalities were attributed to necrotizing fasciitis (NF) in 25 and above‐age populations across the United States (824 in 2003 and 1842 in 2020, a 120.6% increase in total deaths). The overall age‐adjusted mortality rate per 100,000 deaths (AAMR) gradually increased from 0.44 (95% CI: 0.41–0.47) in 2003 to 0.71 (95% CI: 0.68–0.74) in 2020 (Supplement Table 1 and supplement Figure 1).

### 3.1 Necrotizing Fasciitis‐Related AAMR Stratified by Sex

**FIGURE 1:**
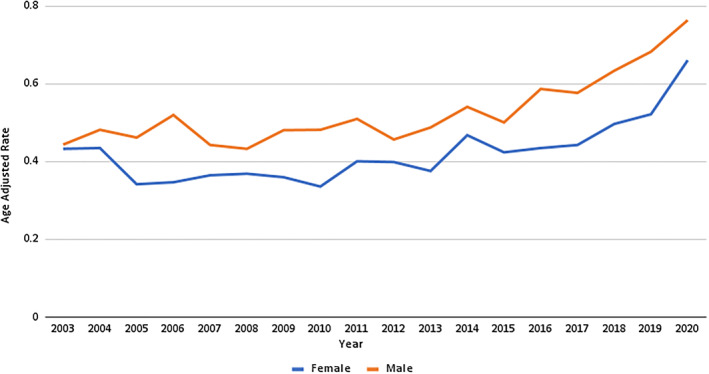
Necrotizing fasciitis‐related age‐adjusted mortality rates per 10,000 stratified by sex in the United States from 1999 to 2020.

The AAMR for males consistently surpassed that of females in all the years (male total AAMR: 0.42; 95% CI: 0.41–0.42 and female total AAMR: 0.37; 95% CI: 0.36–0.38). The male AAMR showed an increase of 0.06 between 2003 and 2015 (APC: 0.98 and 95% CI: −0.1–2.0). After 2015, the AAMR significantly surged from 0.5 to 0.76 by the end of the study period (APC: 8.0 and 95% CI: 4.5–11.5). In contrast, the female AAMR, which was 0.43 in 2003, declined to 0.34 by 2006 (APC: −8.60 and 95% CI: −18.3–2.2). This was followed by a steady increase until 2018 (APC: 2.72 and 95% CI: 1.2–4.1). The remaining period saw a sharp rise, peaking at an all‐time high NF‐related AAMR of 0.66 in 2020 (APC: 17.57 and 95% CI: −0.5–39.0). (Figure 1, Supporting Information S1: Tables 1 and 2).

### 3.2 Necrotizing Fasciitis‐Related AAMR Stratified by Race

**FIGURE 2:**
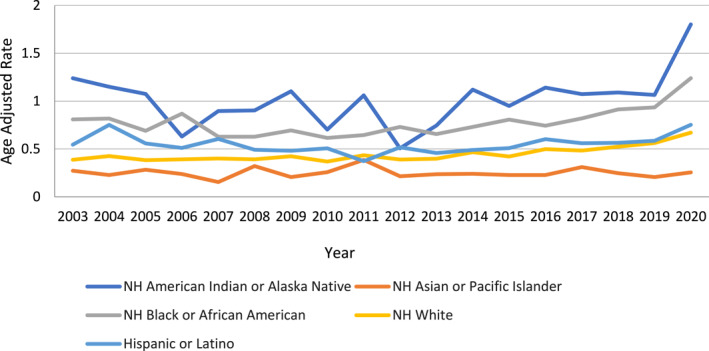
Necrotizing fasciitis‐related age‐adjusted mortality rates per 10,000 stratified by race in the United States from 1999 to 2020.

Among all racial groups, American Indian or Alaska Native patients had the highest AAMR, followed by Black or African American, Hispanic or Latino, White, and Asian or Pacific Islander populations. For the American Indian or Alaska Native group, the AAMR increased steadily from 1.24 in 2003 to 1.80 in 2020. However, no significant trend changes were identified during the study period, as no joinpoint was detected in the APC calculation (APC: 2.26 and 95% CI: −3.3–4.9). In contrast, the AAMR for the Black or African American population initially declined from 0.80 in 2003 to 0.65 in 2013 (APC: −2.12 and 95% CI: −4.7–0.5), but then rose significantly, reaching 1.24 in 2020 (APC: 8.35 and 95% CI: 4.4–12.4). Similarly, the AAMR for the Hispanic or Latino cohort initially decreased from 0.54 in 2003 to 0.37 in 2011 (APC: −5.06 and 95% CI: −8.9 to −1.03), followed by a rise, reaching 0.753 by 2020 (APC: 5.02 and 95% CI: 2.2–7.8). The White population showed a relatively steady increase in AAMR, rising from 0.38 in 2003 to 0.42 in 2015 (APC: 0.74 and 95% CI: −0.5–2.0) and reaching 0.671 in 2020 (APC: 8.42 and 95% CI: 4.2–12.7). The Asian or Pacific Islander population exhibited stable AAMR trends, slightly decreasing from 0.27 in 2003 to 0.25 in 2020 (APC: −0.22 and 95% CI: −2.1–1.7). However, similar to the American Indian or Alaska Native group, no significant trend changes were identified for the Asian or Pacific Islander population, as no joinpoint was detected in the analysis (Figure 2, Supporting Information S1: Tables 2 and 3).

### 3.3 Necrotizing Fasciitis‐Related AAMR Stratified by Region

**FIGURE 3:**
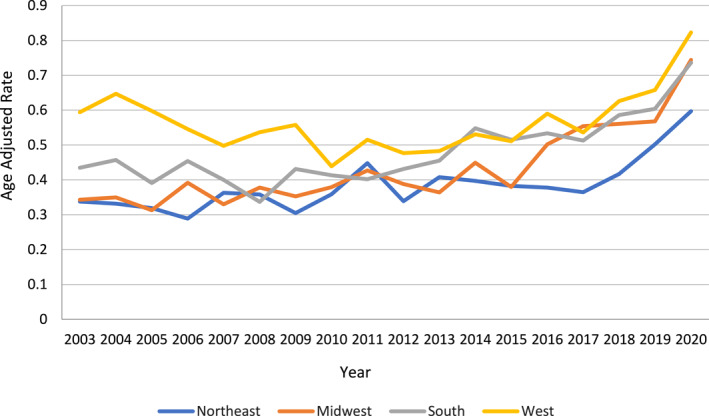
Necrotizing fasciitis‐related deaths stratified by region adults in the United States from 1999 to 2020.

The AAMR across different regions showed significant variations, with the West recording the highest overall AAMR and the Northeast the lowest. In the Northeast, the AAMR rose from 0.33 in 2003 to 0.41 in 2018 (APC: 1.53 and 95% CI: 0.26–0.28) and continued to increase, reaching 0.597 in 2020 (APC: 21.58 and 95% CI: −3.5–53.4). Similarly, the AAMR in the Midwest rose in two stages: a slight increase from 0.34 in 2003 to 0.38 in 2015 (APC: 1.95 and 95% CI: 0.35–3.5), followed by a more significant rise to 0.744 in 2020 (APC: 10.9 and 95% CI: 5.9–16.0). In contrast, the South region's AAMR initially decreased from 0.43 in 2003 to 0.37 in 2008 (APC: −4.18 and 95% CI: −9.8–1.8) before rising to 0.73 in 2020 (APC: 4.95 and 95% CI: 3.5–6.3). In the West, the AAMR declined from 0.59 in 2003 to 0.48 in 2013 (APC: −2.84 and 95% CI: −4.8 to −0.8) and then sharply increased, reaching 0.82 in 2020 (APC: 7.1 and 95% CI: 3.9–10.3). (Figure 3, Supporting Information S1: Tables 2 and 4).

### 3.4 Necrotizing Fasciitis‐Related AAMR Stratified by Urban‐Rural Distribution

Large central metropolitan areas recorded the highest NF‐related AAMR, whereas large fringe regions had the lowest. The AAMR in large central metropolitan areas increased from 0.45 in 2003 to 0.71 in 2020 (APC: 1.32 and 95% CI: 0.1–2.4). However, no joinpoints were identified in the analysis, indicating no statistically significant changes during this period.

In large fringe areas, the AAMR initially declined from 0.37 in 2003 to 0.29 in 2012 (APC: −1.59 and 95% CI: −3.8 to 0.7), followed by a significant increase, reaching 0.593 in 2020 (APC: 5.94 and 95% CI: 3.4–8.4).

Medium metropolitan areas exhibited notable variations in AAMR. Initially, it decreased from 0.478 in 2003 to 0.38 in 2007 (APC: −6.6 and 95% CI: −12.8 to 0.01). This trend then reversed, with the AAMR rising to 0.65 in 2018 (APC: 4.0 and 95% CI: 2.4–5.5) and further increasing to 0.85 by the end of the study period in 2020 (APC: 16 and 95% CI: −0.4–35.4).

**FIGURE 4:**
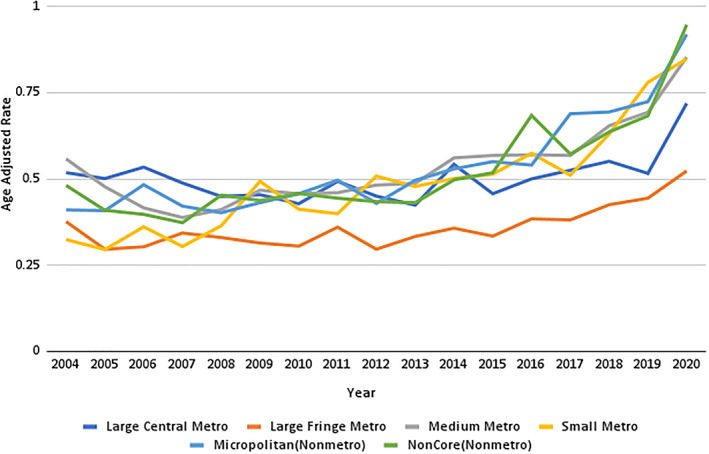
Necrotizing fasciitis‐related age‐adjusted mortality rates per 10,000 stratified by the urban–rural classification in the United States from 1999 to 2020.

Small metropolitan areas recorded an AAMR of 0.48 in 2003, which subsequently declined to 0.29 by 2005 (APC: −18.9 and 95% CI: −48.3 to 27.1). In the following years, the AAMR increased significantly, reaching 0.84 in 2020 (APC: 6.33 and 95% CI: 4.7–7.9). (Figure 4).

**FIGURE 5:**
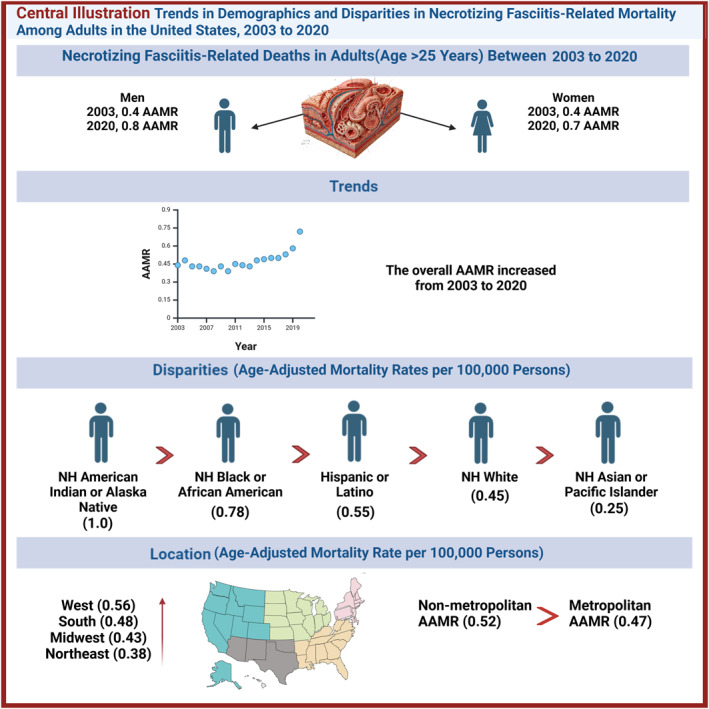
Central illustrations.

Two trends were observed in the AAMR of the micropolitan population throughout the study period. Initially, the AAMR increased slightly from 0.44 in 2003 to 0.49 in 2018 (APC: 1.0 and 95% CI: 3.9–3.0). This was followed by a more substantial rise, culminating in an AAMR of 0.91 by the end of the study period in 2020 (APC: 9.0 and 95% CI: 6.1–11.9). Similarly, the AAMR of noncore area mortality showed an initial increase from 0.40 at the beginning of the study period to 0.43 in 2013 (APC: 0.53 and 95% CI: −2.1–3.3). This upward trend continued until 2020 when the AAMR reached 0.94 (APC: 9.64 and 95% CI: 5.4–13.9). (Figure 5, Supporting Information S1: Tables 1 and 5).

## 4 Discussion

Necrotizing fasciitis is a severe and life‐threatening infection that affects approximately 0.4 individuals per 100,000 people annually in the United States [12]. Our study employed multiple joinpoint models to analyze data from 2003 to 2020 across various U.S. regions and urban–rural areas. This analysis provided valuable insights into the sex, racial, and geographic disparities among populations diagnosed with this debilitating condition (highlighted shows corrections).

Our analysis of mortality data for necrotizing fasciitis from the Centers for Disease Control and Prevention (2003–2020) revealed significant trends. Overall, the age‐adjusted mortality rate (AAMR) remained stable from 2003 to 2013, followed by a gradual increase starting in 2014, culminating in a notable spike from 2019 to 2020. Sex‐stratified results indicated a consistent rise in AAMR among males, with a sharp acceleration observed after 2017. The non‐Hispanic American Indian or Alaska Native populations had the highest AAMR, which exhibited a continuous upward trend. In contrast, the non‐Hispanic Asian or Pacific Islander populations had the lowest rates. Initially, AAMRs by region were comparable; however, by 2015, nonmetropolitan and small‐to medium‐sized metropolitan areas surpassed large central and fringe metropolitan areas in AAMR. Regional variations were pronounced, with the West showing the highest AAMR and the Northeast exhibiting the lowest (Figure 5 central illustration).

In contrast to other diseases where mortality rates have either decreased or stabilized, the mortality associated with necrotizing fasciitis has increased since 2014. This rise can be attributed to various factors, including the growing prevalence of diabetes, obesity, and alcohol use disorders, all of which are established risk factors for necrotizing fasciitis [13]. Additionally, the opioid epidemic may have significantly contributed to the incidence of cellulitis and necrotizing fasciitis among injection drug users [14]. The use of nonsterile needles, combined with poor hygiene and contaminated drug supplies, creates ideal conditions for severe bacterial infections, further increasing the risk [15].

Deaths from opioid overdoses have surged tenfold since 2000, with a major shift in drug use from heroin to synthetic fentanyl around 2014. This shift likely contributed to the rising mortality rates from necrotizing fasciitis, as fentanyl, when injected, is frequently linked to both overdose deaths and severe skin infections [16]. Moreover, the COVID‐19 pandemic worsened this trend in 2020 by reducing access to healthcare and delaying diagnosis, leading to more advanced cases of necrotizing fasciitis and poorer outcomes [17, 18, 19].

This rise in cases has significantly impacted hospitalization rates, particularly in resource‐restricted safety‐net hospitals, where access to emergency surgical management and postoperative care is limited [20]. Additionally, necrotizing fasciitis is commonly misdiagnosed as cellulitis, resulting in delays in surgical treatment and further straining healthcare systems [21, 22]. The need for advanced care, including multiple debridements, is associated with prolonged hospitalization, substantial morbidity, and the need for rehabilitation, highlighting the significant economic burden of the condition [23].

Ethnic disparities in mortality rates were evident. Black or African Americans initially experienced a decrease in mortality that significantly increased in 2014 and continued to rise after that. This trend suggests that Black individuals' lack of insurance and healthcare facilities could have persisted even after the implementation of the Affordable Care Act [24]. In our study, the age‐adjusted mortality rate (AAMR) was higher among Black individuals compared to White individuals, consistent with previous studies indicating higher rates of chronic conditions in the Black population [25, 26]. Additionally, life expectancy is lower for non‐Hispanic Black individuals than for non‐Hispanic White individuals [6]. These disparities in healthcare access, along with delays in diagnosis and disease management, may contribute to the higher mortality rates observed among American Indian and Black populations compared to their White counterparts [27].

Additionally, our study indicates that non‐Hispanic Asians exhibited a consistently low mortality rate for necrotizing fasciitis, which did not increase compared to other racial groups. Our findings are consistent with another study that found that the incidence of necrotizing fasciitis was 28% lower among Asian individuals than among White individuals [7]. This disparity may be multifactorial and could be explained by differences in the mechanisms of organ impairment in septic syndrome [28]. Furthermore, since the mortality rates were not adjusted for socioeconomic status, comorbidities, and health insurance coverage, these factors may also contribute to the observed racial disparities in mortality.

Geographically, the Northeast and Midwest experienced sharp increases in mortality rates following 2015 and 2018, respectively. After an initial decline, the South saw rising age‐adjusted mortality rates (AAMRs) post‐2008, whereas the West witnessed a sudden spike after years of decline. These regional differences are likely influenced by factors such as demographics, ethnic disparities, environmental conditions, healthcare access, and the presence of high‐risk populations. The substantial increase in mortality observed after 2013 may be linked to regions vulnerable to climate change. Significant events, such as Hurricane Sandy in 2013 and Hurricane Harvey in 2017, greatly affected parts of the Northeast and Midwest [29]. Minor injuries during these disasters can serve as entry points for pathogens that cause severe infections such as necrotizing fasciitis. Moreover, exposure to *Streptococcus* pyogenes in floodwaters following hurricanes may contribute to the rising incidence of this “flesh‐eating” disease. Similarly, the western region's vulnerability to wildfires, earthquakes, and droughts may also create an environment conducive to the spread of necrotizing fasciitis [30].

The rising incidence in these areas may be attributed to increased healthcare utilization. This trend follows the expansion of insurance coverage, such as that provided by the Affordable Care Act [31]. The aforementioned policy encouraged more individuals to seek medical care and get diagnosed. However, despite these advancements, income inequality and residential segregation perpetuate significant geographic disparities. Health resources are still unevenly distributed, often mirroring racial and ethnic residential clusters. Although expanded insurance coverage has improved care access, deep‐rooted inequities persist [32].

The concentration of racial and ethnic groups significantly influences necrotizing fasciitis (NF) mortality. The South, which has a large Black population, reports higher NF cases, contributing to its elevated mortality rates. Migration patterns from the South to Midwest cities, such as Detroit and Chicago, reflect this trend as does by the increasing racial diversity in the Northeast. These demographic clusters impact the distribution of NF mortality. Additionally, the South's high prevalence of diabetes—driven by socioeconomic disparities, poverty, and sedentary lifestyles—exacerbates its NF burden [33]. Diabetes, a significant risk factor for NF, contributes to the region's higher disease incidence [34]. Chronic conditions, such as liver disease, may explain the reversals in age‐adjusted mortality rates in these areas. Addressing these health disparities is crucial for reducing NF prevalence [35].

Our study reveals distinct age‐adjusted mortality rate (AAMR) patterns for necrotizing fasciitis across metropolitan and nonmetropolitan areas. Key risk factors include urbanization, population density, healthcare insurance coverage, access to specialized physicians, and proximity to tertiary care centers. Large metropolitan areas, characterized by dense populations and increased environmental exposure, demonstrate a consistent and steady rise in mortality, indicating persistent underlying risks [36]. High population density can lead to sanitation challenges and crowded living conditions, which increase the likelihood of contact, exposure, and transmission of infections [36]. This phenomenon occurs even in regions with relatively better access to healthcare facilities, as the sheer volume of people and environmental factors heighten the risk despite the availability of medical infrastructure.

Improved insurance coverage and new healthcare policies have significantly influenced regional trends in healthcare access. Choi's findings highlight this care gap, revealing that approximately 60% of the uninsured population had little to no physician visits [37]. Lower‐income groups, particularly those earning under 200% of the federal poverty level, are more likely to be uninsured, negatively impacting healthcare access across all regions [37]. In rural areas, persistent poverty and limited healthcare access further contribute to the sharp rise in age‐adjusted mortality rates (AAMRs) [38, 39]. The lack of specialists in these regions hinders timely treatment for necrotizing fasciitis, requiring rapid intervention [40].

This article presents several limitations that should be considered. First, as with many studies relying on ICD coding and death certificate data, there is a potential for misclassification or underreporting of necrotizing fasciitis as a cause of death, which may introduce bias. To capture a broader population, we utilized multiple cause‐of‐death data rather than solely the underlying cause of death, which might result in a less accurate representation of the disease. Second, the dataset lacks detailed clinical information, such as microbiological data, wound culture results, and the extent of tissue involvement. These factors are crucial for understanding the severity and progression of the disease. Third, there is no available data on interventions and treatments for necrotizing fasciitis, limiting our ability to evaluate the impact of various treatment modalities. Finally, the study does not provide information on social determinants of health, including income, education level, and access to healthcare, which could influence disease progression and patient outcomes in cases of necrotizing fasciitis.

## 5 Limitations

This article has a number of limitations to consider. First, as with many studies reliant on ICD coding and death certificate data, there is the potential for misclassification or underreporting of necrotizing fasciitis as a cause of death. This may introduce bias. Second, this dataset lacks detailed clinical information, such as microbiological data, wound culture results, or the extent of tissue involvement, which are important for understanding disease severity and progression. CDC database cannot also provide data on relevant comorbid conditions, which makes it difficult to have a holistic review of the disease. Third, data for intervention and treatment for necrotizing fasciitis were not given, which limits the ability to evaluate the impact of treatment. Finally, information regarding social health determinants, such as income, education level, and access to healthcare, was unavailable, which may influence both disease progression and outcomes for patients with necrotizing fasciitis.

## 6 Conclusion

**FIGURE 6:**
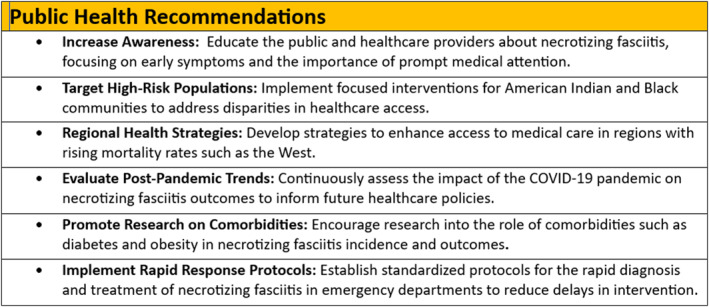
Public health recommendations.

Necrotizing fasciitis, a rapidly progressing and often fatal infection, demands urgent attention as it remains frequently misdiagnosed and underestimated. Our findings highlight a disturbing trend in rising mortality rates, underscored by the interplay of socioeconomic disparities, limited access to healthcare, and the exacerbating effects of climate change. Vulnerable populations, particularly those with chronic conditions, such as diabetes, are disproportionately affected, revealing the urgent need for systemic change. To combat this grave public health challenge, we must prioritize equitable healthcare access, enhance training for healthcare providers, and reinforce public health infrastructures. Raising awareness among the public and healthcare professionals is essential for early detection and intervention. As communities grapple with this life‐threatening disease, a collective commitment to understanding its patterns and enhancing reporting mechanisms will empower us to confront and ultimately reduce the devastating impact of necrotizing fasciitis. It is time to mobilize efforts that transform knowledge into action, ensuring that no individual faces this perilous condition alone (Figure 6).

## Author Contributions

**Saad Khan:** conceptualization, supervision, writing – original draft, writing – review and editing. **Rizwan Ahmad:** writing – original draft, writing – review and editing. **Aqsa Munir:** investigation. **Safa Nasir:** methodology, writing – review and editing. **Maryam Adnan:** writing – original draft, writing – review and editing. **Fatima Naveed:** writing – original draft, writing – review and editing. **Usama Idrees:** writing – original draft, writing – review and editing. **Syeda Mashal Fatima:** writing – original draft, writing – review and editing. **Javed Iqbal:** writing – review and editing.

## Conflicts of Interest

The authors declare no conflicts of interest.

## Supporting information

Supporting Information S1

Figure S1
