# Supplementary material for: Trends in Necrotizing Fasciitis‐Associated Mortality in the United States 2003–2020: A CDC WONDER Database Population‐Based Study
Source: World J Surg. 2025 Mar 19;49(5):1210–8. doi: 10.1002/wjs.12504 (PMC12058447; doi:10.1002/wjs.12504)
Supplement: Supplementary file 1 — Supporting Information S1 [file WJS-49-1210-s002.docx]

**Author’s contribution:**

S.K conceptualized and designed the study. R.A. and A.M., assisted in data acquisition and analysis. S.N. and M.A. contributed to methodology and statistical validation. S.M.F. and J.I. were involved in data curation and visualization. F.N. and U.I. provided oversight and guidance on study methodology and critical review of the manuscript. All authors contributed to drafting and revising the manuscript, approved the final version, and agreed to be accountable for all aspects of the work.

**Acknowledgment:**

##### Open Access funding provided by the Qatar National Library.

**Funding:**

None.

**competing interest:**

No conflict of interest is disclosed by the authors.

**Availability of data and materials:**

No new data was created.

**Figure/table legend**

Figure 1: Necrotizing fasciitis related Age-Adjusted Mortality Rates per 10,000, Stratified by gender in the United States, 1999 to 2020

Figure 2: Necrotizing fasciitis -related Age-Adjusted Mortality Rates per 10,000, Stratified by race in the United States, 1999 to 2020

Figure 3: Necrotizing fasciitis related Deaths, Stratified by region Adults in the United States, 1999 to 2020

Figure 4: Necrotizing fasciitis -related Age-Adjusted Mortality Rates per 10,000, Stratified by Urban-Rural Classification in the United States, 1999 to 2020

Supplemental Figure 1 Age-Adjusted Mortality Rates per 10,000 for Deaths Related to Necrotizing fasciitis

supplemental Table 1: Necrotizing fasciitis related Deaths, Stratified by region Adults in the United States, 1999 to 2020

Supplemental Table 2 : Annual percent change (APC) of Necrotizing fasciitis –related Age-Adjusted Mortality Rates per 10,000 in the United States, 1999 to 2020 maryam

supplemental Table 3: Necrotizing fasciitis- related Age-Adjusted Mortality Rates per 10,000, Stratified by gender in the United States, 1999 to 2020

Supplemental Table 4: Necrotizing fasciitis-related Age-Adjusted Mortality Rates per 10,000, Stratified by race in the United States, 1999 to 2020

Supplemental Table 5: Necrotizing fasciitis-related Age-Adjusted Mortality Rates per 10,000, Stratified by Urban-Rural Classification in the United States, 1999 to 2020
