# Supplementary figures and images for: Trends in Necrotizing Fasciitis‐Associated Mortality in the United States 2003–2020: A CDC WONDER Database Population‐Based Study
Source: World J Surg. 2025 Mar 19;49(5):1210–8. doi: 10.1002/wjs.12504 (PMC12058447; doi:10.1002/wjs.12504)

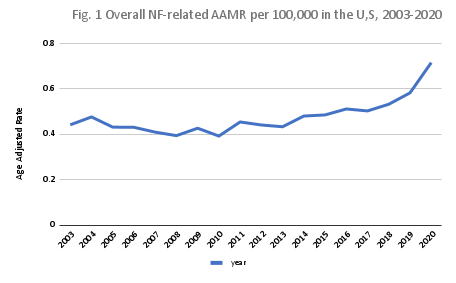

Supplement: Supplementary file 2 — Figure S1 [file WJS-49-1210-s001.png]
